# Intramural Hematoma of Gastrointestinal Tract in People with Hemophilia A and B

**DOI:** 10.3390/jcm12093093

**Published:** 2023-04-24

**Authors:** Wei-Jung Teng, Ching-Huei Kung, Mei-Mei Cheng, Jia-Ruey Tsai, Chia-Yau Chang

**Affiliations:** 1 School of Medicine, College of Medicine, Taipei Medical University, Taipei City 110, Taiwan; weizonten@gmail.com; 2 Department of Medical Imaging, Taipei Medical University Hospital, Taipei City 110, Taiwan; 3 Division of Pediatric Gastroenterology, Department of Pediatrics, Cheng Hsin General Hospital, Taipei City 112, Taiwan; 4 Department of Hematology and Oncology, Taipei Medical University Hospital, Taipei City 110301, Taiwan; 5 Hemophilia Center, Taipei Medical University Hospital, Taipei City 110301, Taiwan; 6 Department of Pediatrics, School of Medicine, College of Medicine, Taipei Medical University, Taipei City 110, Taiwan; 7 Division of Pediatric Hematology and Oncology, Department of Pediatrics, Taipei Medical University Hospital, Taipei City 110301, Taiwan

**Keywords:** hemophilia, intramural hematoma, conservative treatment, clotting factor concentrates, factor VIII inhibitor

## Abstract

People with hemophilia (PWH), especially severe hemophilia, often experience bleeding episodes, which occur mostly at major joints. Intramural hematoma of the gastrointestinal (GI) tract is a rare, potentially life-threatening clinical bleeding manifestation in PWH. Prompt identification and timely administration of clotting factor concentrates are of utmost importance for effective management and optimal patient outcomes. In this report, we present the case of a 48-year-old male with severe hemophilia A. The patient developed a spontaneous intramural hematoma of the jejunum, leading to signs of acute abdomen, bloody stool, and paralytic ileus. Conservative management with factor VIII (FVIII) infusion was successfully administered. However, within a span of three months, the patient suffered from a recurrent episode of intramural hematoma, which was again effectively treated with conservative therapy. Subsequently, prophylactic FVIII therapy was administered to the patient, resulting in the absence of recurrence for over three years. Inspired by this case, we conducted a comprehensive review of the relevant literature and gathered data from 79 reported cases of intramural hematoma that were documented between the years 1956 and 2022. We classified these cases based on the site affected within the gastrointestinal (GI) tract (spread across five different locations) and proceeded to conduct a simple pooling analysis on the data collected, which subsequently revealed that the overall mortality rate of intramural hematoma in people with hemophilia (PWH) was found to be 12.2%, while children have a higher mortality rate (23.3%) than adults (4.9%). We hope this case report and literature review increase awareness of this rare bleeding manifestation in PWH, the effectiveness of conservative treatment, and the possibility of prophylaxis against recurrence.

## 1. Introduction

Hemophilia A and B are hereditary bleeding disorders characterized by deficiencies in coagulation factor VIII (FVIII) and factor IX (FIX), respectively [1,2]. Individuals with severe forms of these disorders, where the endogenous level of clotting factor is less than 1%, are prone to hemorrhage in various sites, such as the joints, muscles, soft tissues, central nervous system, or genitourinary tract [2,3]. Major joint bleeds are the most common manifestation. To manage bleeding in these patients, replacement therapy with FVIII or FIX is required.

Gastrointestinal (GI) bleeding, characterized by bleeding into the lumen of the GI tract, is a frequent complication among people with hemophilia (PWH), with a reported incidence ranging from 10% to 25% [4,5]. However, PWH rarely experience bleeding into the submucosal layer of the GI tract, which is known as intramural hematoma. Furthermore, recurrent cases of intramural hematoma in PWH are even more infrequent [3,4,5,6].

### 1.1. Brief Introduction of Intramural Hematoma

The first case of intramural intestinal hematoma was reported by McLouchlan in 1838, and it took an additional 100 years for Liverud to provide the first radiological description of this condition [7,8]. Intramural hematoma is chiefly prevalent in children who experience blunt trauma [9].

However, nontraumatic or spontaneous intramural hematoma may occur in patients with impaired coagulation, in which anticoagulant therapy represents the primary cause in most cases. Additionally, spontaneous intramural hematoma is also observed in individuals with other bleeding disorders, such as idiopathic thrombocytopenic purpura and von Willebrand disease. Pancreatic diseases, liver failure, leukemia, lymphoma, and vasculitis are among the other risk factors for intramural hematoma [7,10,11].

### 1.2. Main Structure of Our Review—Started from One Real-World Case

In this report, we first present the case of a 48-year-old male with severe hemophilia A. The patient experienced a spontaneous intramural hematoma of the jejunum, which resulted in acute abdomen, bloody stool, and paralytic ileus. Despite being considered for surgical intervention twice, conservative management utilizing factor VIII (FVIII) infusion was successfully administered, and the patient’s condition demonstrated a favorable outcome. However, within three months, recurrent episodes of intramural hematoma occurred, prompting repeat use of FVIII infusion, which ultimately led to a full recovery. Following this, prophylactic FVIII therapy was given to the patient, resulting in the absence of any hematoma recurrence for over three years.

Further, we conducted a comprehensive review of 79 reported cases of intramural hematoma in patients diagnosed with hemophilia A or hemophilia B. The analysis of these cases was based on the specific location of the hematoma within the gastrointestinal (GI) tract, leading to the identification of five distinct groups. In order to provide insights into the clinical presentation, diagnosis, management, and outcomes of these hematoma cases, a simple pooling analysis was conducted with a particular emphasis on delineating differences between the various sites within the GI tract where hematomas occurred. Notably, our findings led us to recommend conservative treatment as the primary therapeutic option over surgical intervention.

## 2. Case Description

A 48-year-old male who suffers from severe hemophilia A with a FVIII activity of less than 1%. He had been receiving on-demand therapy with FVIII infusion for over 40 years and has been under follow-up care at our hospital for 15 years. FVIII inhibitor had never been detected. He suffers from severe arthropathy affecting the right hip, left knee, and both ankles. Additionally, he has a history of hepatitis C virus (HCV) infection, which was treated successfully under anti-HCV therapy without virus detection for more than 15 years.

**Figure 1:**
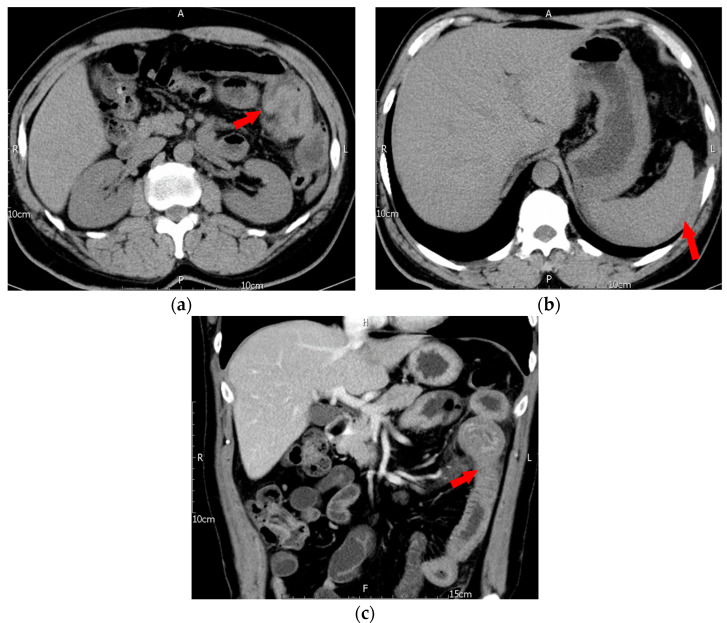
First episode of intramural hematoma of jejunum. (**a**) Axial non-contrast CT showed marked segmental wall thickening of the proximal jejunum with diffuse hyperdensity (arrow), (**b**) axial non-contrast CT showed perisplenic hemoperitoneum (arrow), (**c**) coronal reformatted contrast-enhanced CT showed contrast enhancement of the inner mucosa, but there was no contrast enhancement of the thickened wall of the corresponding proximal jejunum, which indicated the presence of an intramural hematoma (arrow).

In late June 2019, this patient, with a body weight of 70 kg, was admitted to the emergency department with a complaint of generalized abdominal pain that had persisted for 3 days. The pain was mainly located on the left side and in the periumbilical regions. Associated symptoms included decreased appetite and nausea, but the patient did not have a fever. The patient had experienced tarry stool one day before admission. The patient had no history of trauma. On physical examination, the patient had periumbilical tenderness and rebound tenderness. Lab data revealed leukocytosis (WBC: 13,170/µL) with neutrophil predominance, an elevated C-reactive protein (CRP) level of 5.09 mg/dL, and a normal hemoglobin (Hb) level (Hb: 14.9 g/dL). Platelet count was normal at 398 × 10^3^/µL. His prothrombin time/international normalized ratio (PT/INR) was normal at 1.14 but activated partial thromboplastin time (aPTT) was prolonged at 146.3 s (control: 32–45 s). Under the suspicion of acute abdomen, a computed tomography (CT) of the abdomen was performed, revealing bowel wall thickening in a wide segment of the jejunum, consistent with an intramural hematoma and an intussusception-like lesion in the jejunal region. Further, there was ascites with hyperdense layering noted in the perisplenic and subphrenic areas and the cul-de-sac, consistent with hemoperitoneum (Figure 1).

Following hospitalization, the patient was placed on bowel rest and administered regular infusions of rFVIII-Fc (Eloctate, an extended-half-life recombinant FVIII, 1000 IU q12h, 28.5 IU/kg/day). A pharmacokinetic study of Eloctate calculated a half-life of 24.75 h from medical records and the Web-Accessible Population Pharmacokinetic Service–Hemophilia (WAPPS-Hemo) online system [12]. Maintenance of trough levels of FVIII:C at approximately 40% (using one-stage assay) was monitored. Antibiotic therapy with Flomoxef was administered for potential infection control. A surgical doctor was consulted regarding suspected intussusception and suggested diagnostic laparoscopy in case of persistent bloody stool. Pain levels progressively decreased upon administration of FVIII infusion, and tarry stool was no longer detected. As a result, FVIII dosing was gradually tapered to 1000 IU qd, with the introduction of a clear liquid diet and subsequent transition to a soft diet. After 6 days of hospitalization, the patient stabilized without surgical intervention and was discharged without complications. Prophylactic therapy with FVIII was advised. However, due to the patient’s lack of bowel symptoms, the therapy was refused.

**Figure 2:**
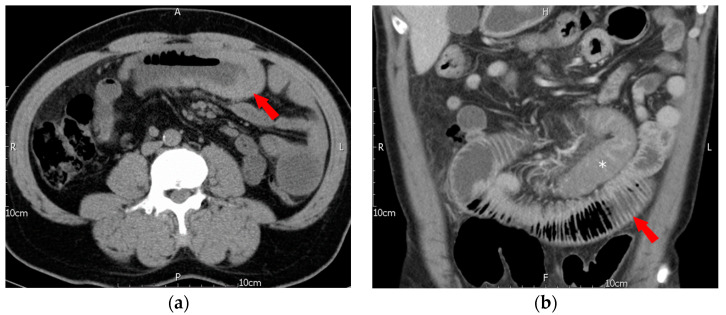
Second episode of intramural hematoma of jejunum. (**a**) Axial non-contrast CT showed segmental thickening of the midjejunum with diffuse hyperdensity, indicating intramural hematoma (arrow), (**b**) coronal reformatted contrast-enhanced CT showed intramural hematoma (*) of the midjejunum and marked dilatation of the proximal jejunum, indicating mechanical ileus (arrow).

However, just two and a half months later, in early September 2019, he presented once again at our emergency department with similar symptoms. This time, the chief complaint was lower and periumbilical abdominal pain that had persisted for 3 days, along with nausea and decreased appetite. The patient had not passed stool for 3 days and had not experienced fever, diarrhea, or tarry or bloody stool recently. Physical examination identified periumbilical tenderness and rebound pain. Lab data indicated leukocytosis (WBC: 13,690/µL) with neutrophil predominance and an elevated CRP level of 6.19 mg/dL. The patient’s Hb level was 15.0 g/dL, and his platelet count was 418 × 10^3^/µL. Although the PT/INR was normal at 1.04, the aPTT was prolonged at >180.0 s (control: 32–45 s). An abdominal CT revealed segmental wall thickening at the midjejunum, compatible with intramural hematoma, causing partial obstruction of the bowel, dilatation of the proximal jejunum and stomach, indicating mechanical ileus. The involved segments of the hematoma differed from those in the previous episode (Figure 2).

After admission, conservative treatment with rFVIII-Fc in the form of Eloctate was administered at a dose of 2500 IU q12h (equivalent to 71.4 IU/kg/day) in order to maintain FVIII trough levels at around 80%. An enteroscopy was carried out during this hospitalization in order to exclude the presence of a jejunal tumor, which yielded negative tumor findings. Nevertheless, the procedure revealed segmental reddishness and swelling of the jejunum with lumen narrowing, compatible with intramural hematoma. Gradual reductions in abdominal pain levels were observed, and a repeated CT scan on the fifth day of hospitalization also indicated the resolution of hematoma and the absence of any evidence of small bowel obstruction. After a period of 9 days of hospitalization, the patient’s condition stabilized, and he was subsequently discharged without any complications. He received prophylactic treatment at home with Eloctate at a dose of 4500 IU once per week (equivalent to 64.3 IU/kg/week) in order to maintain FVIII trough levels at around 2–4%. No recurrences of intramural hematoma were recorded during the three-year period following the patient’s discharge (up to the point at which this report was written).

## 3. Materials and Methods

### 3.1. Data Sources, Searches, and Study Selection

**Figure 3:**
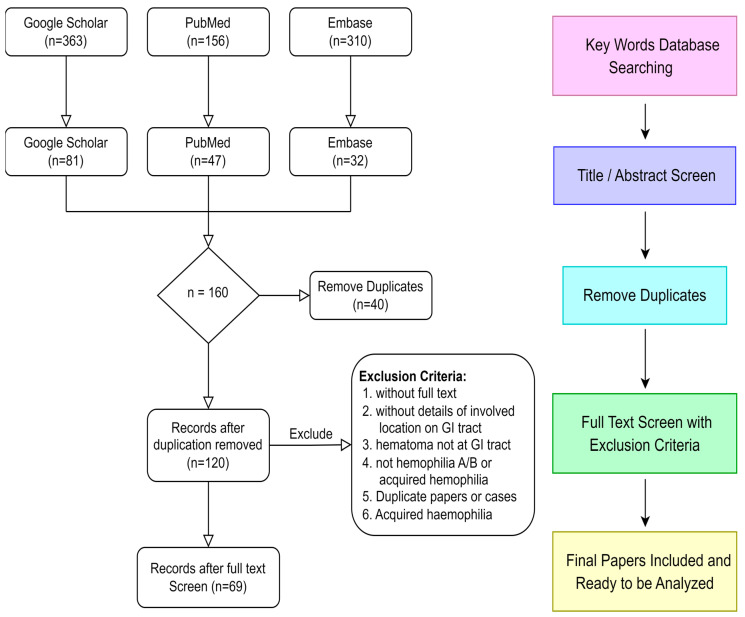
Flowchart of paper selection.

To enhance our understanding of intramural hematoma of the GI tract in PWH, we conducted a literature search on PubMed, Google Scholar and Embase, utilizing the keywords “hemophilia” or “haemophilia”, and “intramural hematoma” or “intramural hemorrhage.” Inclusion criteria were individuals with hemophilia A and hemophilia B, and exclusion criteria were studies without full text or patient details. In the end, we extracted 69 articles, which described a total of 79 cases, including our own, from 1956 until October 2022. The selection process is illustrated in Figure 3.

### 3.2. Clinical Parameters and Statistical Analysis

Data were gathered on several factors, including age, sex, hemophilia subtype, titers of clotting factor inhibitors, whether prophylactic treatment was administered prior to the bleeding event, and any relevant trauma history. Clinical features of complications such as nausea or vomiting (possible signs of ileus or GI obstruction), tarry or bloody stool or hematemesis (possible signs of bleeding into the lumen of the GI tract), hemoperitoneum (possible sign of bleeding out of the serosa of the GI tract), and intussusception were recorded if reported in the research. However, if a paper did not report these symptoms, the patients were assumed not to have them. Laboratory data, including Hb levels and leukocyte counts, were also collected. Clinical management, classified as either operative or conservative therapy, was examined. The primary outcome was either recovery (symptoms subsided or hematoma resolved) or mortality.

A simple pooling analysis was conducted, and statistical relationships among the above-mentioned parameters were analyzed using Pearson’s chi-square test or Fisher’s exact test for categorical data, while Wilcoxon rank-sum test was used for continuous data. A *p*-value of less than 0.05 was considered significant. All statistical analyses were performed using the SAS 2.12.0 software from the Free Software Foundation in Boston, MA, USA.

### 3.3. Limitations

Nonetheless, caution must be exercised when interpreting these results, as several limitations need to be considered. The primary constraint pertains to the fact that the investigation encompassed articles published from 1956 to 2022, spanning a duration of 66 years, wherein the diagnostic instruments and medical interventions underwent considerable fluctuations. A second notable limitation is the inability to examine the regimen and utilization of factor replacement therapy in surgery and conservative management due to the shortage of relevant data. Nevertheless, it is important to consider that the brands, regimen, and dosing frequency for clotting factor concentrates (CFCs) infusion may substantially impact clinical outcomes. The third limitation concerns clinical parameters that were not included in the literature, potentially confounding the statistical findings. For instance, if the paper failed to report a particular symptom or complication, we labeled it as “symptom negative” or “complication negative”, which may have led to an over or underestimation of clinical outcomes. Fourth, the possibility of excluding pertinent literature cannot be ruled out. Despite meticulous scrutiny of the literary sources in the three designated databases, the elimination of studies without complete text and comprehensive patient information may have resulted in the absence of valuable data. It should be noted that most of the literature incorporated in this study was composed in English, increasing the possibility of overlooking relevant literature in other languages.

Notwithstanding, our collection of 79 cases from 69 papers, with no artificial selection bias, should provide some degree of representation across various ages. For instance, approximately 30% of the 79 cases involved inhibitor patients with intramural hematoma, which corresponds to the prevalence of inhibitor patients in PWH. Consequently, our findings and analysis should reflect the real-world setting to some extent.

## 4. Results

### 4.1. Clinical Characteristics of the Overall Selected Cases

Among the 79 cases extracted from 69 papers, data on sex was available for 72 cases, 72 of which were men, and none were women. Age information was given for 76 cases, where 31 of them were children under the age of 18 and 45 were adults. Hemophilia A was present in 62 cases, hemophilia B in 4 cases, and 13 cases were of an unknown hemophilia type. Of the 62 patients with hemophilia A, 23 had severe hemophilia, 7 had moderate hemophilia, 5 had mild hemophilia, and 27 had an unknown severity of hemophilia.

### 4.2. Prevalence of Intramural Hematoma in Hemophilia A and B

In our research, the recorded ratio of cases of hemophilia A to hemophilia B was 62:4 (31:2). However, the prevalence of hemophilia A and B is estimated to be approximately 1/10,000 and 1/50,000, respectively, resulting in a ratio of approximately 5:1. Our review of the data indicates that intramural hematoma occurs more frequently in patients with hemophilia A than in those with hemophilia B. To the best of our knowledge, this finding has not been previously reported. Although the bleeding phenotypes of hemophilia A and hemophilia B have been thought of as highly similar, some authors have argued that the bleeding tendency of hemophilia B may be less severe than that of hemophilia A and thus that hemophilia B has superior long-term outcomes [13,14,15]. This may be compatible with the lower rate of intramural hematoma observed in patients with hemophilia B as compared to those with hemophilia A. However, the underlying mechanism responsible for this difference remains unclear. One possible factor may be the unique characteristics of FIX extravasation with collagen IV binding, which could lead to different bleeding tendencies in patients with hemophilia A and B [16].

### 4.3. Comparisons between Inhibitor and Non-Inhibitor Groups

Inhibitor formation is the most serious complication of CFCs replacement therapy for PWH. The inhibitor prevalence in people with hemophilia A has been reported to be 24–44.5% [17,18]. In our literature review, 46 PWH had inhibitor status details, including 34 (73.9%) patients without inhibitors and 12 (26.1%) patients with inhibitors.

Patients with inhibitors, regardless of their titer levels, were significantly more likely to experience bleeding into the gastrointestinal (GI) lumen than those without inhibitors (*p* = 0.00595 **) and bleeding through the serosa of the bowel into the peritoneum (*p* = 0.0039 **) than those without inhibitors. However, there was no significant difference in operation and mortality rates between patients with and without inhibitors.

It is well-established that patients with inhibitors, particularly those with high-titer inhibitors, are at increased risk of life-threatening bleeding [18,19]. Our results are consistent with this fact, but our analysis also indicated that, with timely diagnosis and proper management, the difference in outcomes between patients with and without inhibitors is not significant.

### 4.4. Clinical Features of Different Involved Sites

Intramural hematoma can occur in any part of the gastrointestinal tract. Of the 79 cases analyzed, the small intestine was involved in 34 cases, the stomach in 10 cases, the esophagus in 3 cases, the duodenum in 15 cases, and the colon in 17 cases. The clinical features of these five sites are presented in Table 1.

The clinical symptoms of gastrointestinal (GI) obstruction, such as nausea and vomiting, were frequently observed in patients with intramural hematoma and were associated with the site of involvement. Specifically, patients with hematoma involving the upper GI tract, including the stomach (8 of 10, 80%), duodenum (8 of 11, 72.73%), and small intestine (22 of 33, 66.67%), were more likely to experience these symptoms compared to those with colonic involvement (5 of 19, 26.3%). This association was found to be statistically significant (*p* = 0.0294 *).

Bleeding into the lumen was more commonly observed in patients with involvement of the small intestine (16 of 33, 48.5%), colon (7 of 16, 43.8%), or esophagus (2 of 3, 66.7%), and less frequently observed in those with involvement of the duodenum (1 of 11, 9.1%) or stomach (1 of 10, 10%). This association was also statistically significant (*p* = 0.0281 *).

On the other hand, neither hemoperitoneum nor perforation of the serosa of the GI tract showed a significant association with the site of involvement (*p* = 0.2901). However, hemoperitoneum or perforation into the peritoneal space was associated with operation, with cases presenting with hemoperitoneum (15 of 25, 60%) being more likely to require operation compared to those without hemoperitoneum (5 of 45, 11.1%) (*p* < 0.0001 ***). The patients with hemoperitoneum (bleeding out of serosa of the GI tract) seemed to have higher mortality than those without hemoperitoneum, but the associated did not achieve significance (*p* = 0.1087).

Mortality was found to differ significantly with the site of involvement (*p* = 0.0076 **), with the highest mortality rates observed in cases involving the duodenum. However, selection bias may have occurred because four of the nine deaths were reported in the same paper, in which only cases with duodenum involvement were reported, and all were pediatric patients [20]. Additionally, the most commonly involved sites differed between children and adults (*p* = 0.0006 ***), with the duodenum (12 of 31, 38.7%) and colon (8 of 31, 25.8%) being more commonly involved in children and the small intestine (26 of 45, 57.8%) being the most involved site in adults.

### 4.5. Comparison between Children and Adults

According to the relevant data for both children and adults, children exhibit a higher prevalence of anemia (defined as a hemoglobin level of less than 11 g/dL), compared to adults, with a prevalence of 81% in children versus 52.8% in adults (*p* = 0.0334 *). In addition, children are more likely to have a history of traumatic events, with a prevalence of 47.4% in children versus 3.4% in adults (*p* = 0.0004 ***). The comparison of the clinical data between children and adults is presented in Table 2.

Furthermore, when comparing continuous Hb levels to the presence versus absence of categorical anemia, the results demonstrate even greater statistical significance (*p* = 0.0074 **), with children displaying lower median Hb levels of 8.1 g/dL compared to adults at a median of 10.7 g/dL. Moreover, unfavorable outcomes were more prevalent in children, with a markedly increased mortality rate of 23.3% (7 out of 30 cases) compared to adults at a rate of 4.9% (2 out of 41 cases) (*p* = 0.0305 *).

### 4.6. Comparison between Recovery and Mortality groups

Of the 74 cases with the prognosis being reported, 9 involved mortality, giving an overall mortality rate of 12.2%. The comparison of the clinical data between the recovery and mortality groups is presented in Table 3. In terms of bleeding into the GI tract, bleeding out of the GI tract’s serosal layer, as well as intussusception, no significant differences were observed between the recovery and mortality groups.

Operations were conducted on a total of 24 patients, out of which only 22 patients reported their prognosis. For the remaining 52 patients, all cases reported their prognosis and were treated with conservative management using clotting factor concentrates infusion (CFCs). The mortality rate was 22.7% (5 cases) among the 22 cases that underwent operations, whereas only 7.7% (4 cases) of the 52 patients treated with conservative management died. However, this difference in mortality rates was not statistically significant (*p* = 0.1149).

Among the nine mortality cases, four of them were reported in the same paper, and the details about the symptoms were not provided but only stated that duodenal involvement was observed [20]. In contrast, among the remaining five mortality cases, three showed reduced hemoglobin levels [11,21,22], and five of them demonstrated signs of bleeding into the lumen or out of the serosa of the gastrointestinal tract (significant hematemesis, bleeding per rectum, or hemoperitoneum) [11,21,22,23,24], two had peritoneal signs [21,23], and two had firm palpable masses on physical examination [21,23].

### 4.7. Clinical Features of Intramural Hematoma-Related Intussusception

Intussusception was observed in 12.7% (10 of 79) of cases, occurring exclusively in the colon (4 cases) and small intestine (6 cases). There was no significant difference in incidence between children and adults, with four cases in children and six cases in adults. Three symptoms were common in cases of intussusception, namely, abdominal pain (all 10 cases), vomiting (9 cases), and bloody stool (5 cases), accompanied by palpable tender masses (5 cases) and paralytic ileus (4 cases).

In terms of management, six cases were treated conservatively, resulting in successful self-reduction within a week [1,25]. The remaining four cases underwent surgical management, including hematoma evacuation and intussusception reduction (1 case) and bowel resection with end-to-end anastomosis (3 cases). The patients in all 10 cases survived and recovered fully without sequelae. Notably, patients with intussusception did not exhibit a higher incidence of operative intervention, bleeding into or out of the gastrointestinal tract, or mortality when compared to those without intussusception.

## 5. Discussion

Intramural hematoma of the gastrointestinal (GI) tract is a rare condition in PWH, despite their bleeding tendency. Bleeding into the walls of the esophagus, stomach, small intestine, or colon has been reported [4,6,26,27]. A hematoma located in the bowel wall can lead to a sudden onset of acute abdominal pain, and patients may present with signs of GI obstruction [3,20]. Consequently, this condition can often be misdiagnosed as acute abdomen and treated with unnecessary laparotomy [2,3,28]. Complications of intramural hematoma of the GI tract include acute intestinal obstruction, paralytic ileus, intussusception, and rupture of the hematoma into the bowel lumen or retroperitoneal space [3,29]. In cases where the hematoma affects the duodenum, obstruction of the ampulla of Vater may cause jaundice and obstructive pancreatitis [20,30,31,32].

### 5.1. Pathological Mechanism

The most common pathological mechanism of intramural hematoma should be the tearing or rupture of terminal arterial vessels leaving the mesentery to penetrate the muscularis of the intestinal wall [4,7,33]. As the hematoma forms, an osmotic gradient within the wall gradually increases, leading to the gradual separation of the muscularis mucosa from the muscle layer [4,7,34]. This may, in turn, result in paralytic ileus and narrowing or obstruction of the GI lumen [35]. Additionally, leakage of blood from a thickened, engorged, or ischemic bowel wall can also occur, leading to the accumulation of hemorrhagic ascites [7,34].

### 5.2. Clinical Manifestations

The most frequently reported clinical manifestation among the cases was abdominal pain, which was mentioned in 61 cases (77.2%). Signs of gastrointestinal obstruction, such as nausea, vomiting, or decreased appetite, were also commonly observed (43 cases). Other clinical manifestations included constipation (7 cases), hematemesis (6 cases), tarry or bloody stool (21 cases), and diarrhea (4 cases). Physical examination revealed abdominal distention in 22 cases, palpable mass in 11 cases, and hypoactive bowel sounds in 10 cases. Hemodynamic instability was identified in cases with severe blood loss. Signs of peritoneal irritation were suggestive of severe conditions, such as bowel wall necrosis, perforation, or hemoperitoneum [1,34]. In cases involving hematoma of the esophagus, dysphagia, chest pain, and hematemesis were reported [36].

### 5.3. Laboratory Findings

Laboratory investigations failed to provide specific diagnostic information regarding intramural hematoma in the patients under study. In 36 cases, reduced levels of Hb were observed, indicative of blood loss. Leukocytosis with a left shift and elevated CRP levels were also present in some cases, which might be associated with hemoperitoneum, more severe intramural hematoma, or even perforation into the lumen or out of the serosa of the GI tract. Elevated amylase and lipase levels are indicators of associated obstructive pancreatitis, especially in cases affecting the duodenum [15,32]. Glassman et al. reported a case of acute obstructive pancreatitis with elevated amylase due to intramural duodenal hematoma. They concluded that an initial conservative approach was recommended instead of surgical management, especially in patients with hemophilia due to their heightened risk of bleeding [32].

### 5.4. Image Findings

Among imaging studies, plain abdominal radiography is nonspecific and may reveal only evidence of GI obstruction if present [7,20,34]. Abdominal ultrasonography is effective in evaluating the presence of free air in the abdominal cavity. Nevertheless, abdominal CT is the definitive diagnostic modality [4,7,20]. It can not only identify the site and extent of hematoma but also reveal potential complications, such as intussusception, local abscess, or perforation [7,20,37]. The use of noncontrast CT is preferred due to intravenous contrast medium obscuring hyperdensity within the bowel wall [38]. Possible CT findings include circumferential wall thickening, hyperdensity in submucosal areas, narrowing of the bowel lumen, GI obstruction, and hyperdense ascites [34].

### 5.5. Intramural Hematoma-Related Intussusception

Intussusception is rare in adults, with only 2–3 cases per million per year [28]. Approximately 90% of such cases exhibit a pathological lead point. Therefore, management of adult intussusception is always surgical [1,37]. Intramural hematoma in PWHs can be a lead point of intussusception. Such patients usually present with acute abdomen, and consequentially, surgical management is often the diagnostic approach [28].

While intussusception is typically treated through surgery, our research indicates that for these PWH, intussusception can be a transient condition that can be resolved through CFC infusion treatment, thereby rendering surgical intervention unnecessary [1,28,39] unless the patient has hemoperitoneum or hemodynamic instability [40].

## 6. Future Directions

### 6.1. Management for Intramural Hematoma in PWH

Rapid and accurate diagnosis, along with timely management, are crucial for PWH with intramural hematoma [1,33]. Conservative therapy with CFCs infusion has been found to be effective, even for those with intussusception or hemoperitoneum [1,6,11,28]. Surgical intervention, in contrast, should be reserved for individuals who are at a higher risk of severe complications, such as intestinal ischemia, bowel perforation, peritonitis, intraperitoneal hemorrhage, intractable intestinal obstruction, or irreversible intussusception [4,7,34,41].

Although perforation into the peritoneal space or hemoperitoneum is typically viewed as requiring immediate surgical intervention, Janic et al. and Benjamin et al. have reported cases of successful nonsurgical management, implying that conservative therapy is a suitable option for PWH with mild hemoperitoneum [6,42]. In cases where surgical intervention is necessary, simple hematoma evacuation is recommended over bypass surgery [20], which should be reserved only for individuals exhibiting perforation or severe intestinal injury [34].

Intriguingly, literature reviews of cases where individuals experienced an anticoagulant overdose and developed intramural hematoma have yielded similar results. The researchers inferred that the majority of such instances can be managed conservatively without the need for surgery [43].

### 6.2. Recurrence and Prophylaxis

Regarding the issue of recurrence, Katsumi et al. reported the case of a 17-year-old patient with severe hemophilia A [5]. This patient, having a high FVIII inhibitor titer (1024 BU/mL), had at least four episodes of intramural small intestinal hematoma recurrence within 54 months, all of which involved the jejunum. The patient received successful conservative management with activated prothrombin complex concentrate along with recombinant activated FVII for each episode. However, it was not indicated whether the patient received prophylactic therapy after the episodes.

In contrast, our patient, a 48-year-old with no inhibitors, experienced one recurrent episode, and subsequently received prophylactic therapy once weekly, and showed no recurrent episodes for the following three years. Given the potentially life-threatening nature of intramural hematoma for PWH, prophylactic therapy with CFCs is crucial for reducing the risk of recurrence after one or more episodes. Prophylactic therapy with regular CFC infusion is effective in significantly reducing the likelihood of spontaneous bleeding events in PWH by reducing disease severity through maintenance of trough factor levels of >1% [44,45,46].

However, complete blocking of recurrent intramural hematoma in PWH with prophylactic therapy is not guaranteed. For example, Prieto et al. reported a 22-year-old patient with severe hemophilia A, who received prophylaxis using FVIII 40 IU/kg three times per week from the age of 19 years and still experienced spontaneous intramural hematoma of the jejunum [4]. In our review, among 35 cases of hemophilia A of known severity, seven were moderate, and five were mild. Nevertheless, prophylaxis can reduce the overall bleeding risk of PWH, including the risk of intramural hematoma. Further case studies are warranted to identify a method for the complete prevention of recurrent intramural hematoma in PWH.

### 6.3. For More Detailed Information

To provide further information, this report showcases all 79 cases of intramural hematoma of the GI tract, along with details pertaining to age, diagnosis, location, symptoms, management, and outcomes. These details are organized according to the specific sites involved: Small intestine in Table 4, stomach in Table 5, esophagus in Table 6, duodenum in Table 7, and colon in Table 8.

## 7. Conclusions

Intramural hematoma of the GI tract is rare among PWH. The presenting symptoms can vary depending on the site of involvement, ranging from mild abdominal pain to severe ileus and even large bloody stools, hemoperitoneum with unstable hemodynamics, and significant hematemesis. The recommended diagnostic choice is abdominal CT without contrast. The overall mortality rate is found to be 12.2%, while children have a higher mortality rate (23.3%) than adults (4.9%). Early conservative therapy with CFCs infusion should be the first-line therapeutic option, and surgical intervention should be reserved for complicated cases. Prophylaxis with regular CFCs infusion to prevent the recurrence of intramural hematoma may be an effective and practicable strategy but does not guarantee complete freedom from recurrence. We believe that our case report with a literature review will be clinically helpful for physicians to manage better the rare risky intramural hematoma in PWH.

## Figures and Tables

**Table 1:** Clinical variables for different involved sites within the GI tract.

Clinical Variables (Case Number)	Esophagus (n = 3)	Stomach (n = 10)	Duodenum (n = 15)	Small Intestine (n = 34)	Colon (n = 17)	*p*-Value
Age ^†^ Children (31) Adult (45)	2 0 (0%) 2 (100%)	10 5 (50%) 5 (50%)	15 12 (80%) 3 (20%)	32 6 (18.8%) 26 (81.2%)	17 8 (47.1%) 9 (52.9%)	0.0006 ***
Nausea/Vomiting ^†^	3	10	11	33	17	0.0294 *
No (30) Yes (44)	2 (66.7%) 1 (33.3%)	2 (20%) 8 (80%)	3 (27.3%) 8 (72.3%)	11(33.3%) 22(66.7%)	12 (70.6%) 5 (29.4%)	
Anemia/Pale ^†^ No (22) Yes (36)	2 0 (0%) 2(100%)	6 1 (16.7%) 5 (83.3%)	8 1 (12.5%) 7 (87.5%)	28 16 (57.1%) 12 (42.9%)	14 4 (28.6%) 10 (71.4%)	0.0550 *
Bleeding into lumen of GI tract ^†^ No (46) Yes (27)	3 1 (33.3%) 2 (66.7%)	10 9 (90%) 1 (10%)	11 10 (90.9%) 1 (9.1%)	33 17 (51.5%) 16 (48.5%)	16 9 (56.2%) 7 (43.8%)	0.0281 *
Bleeding out of serosa of GI tract ^†^ No (47) Yes (26)	3 2 (66.7%) 1 (33.3%)	10 7 (70%) 3 (30%)	11 4 (36.4%) 7 (63.6%)	33 24 (72.7%) 9 (27.2%)	16 10 (62.5%) 6 (37.5%)	0.2901
Intussusception ^†^ No (68) Yes (10)	3 3 (100%) 0 (0%)	10 10 (100%) 0 (0%)	15 15 (100%) 0 (0%)	33 27(81.8%) 6 (18.2%)	17 13(76.5%) 4 (23.5%)	0.1783
Management ^†^	3	10	15	32	16	0.0221 *
Conservative (52) Operation (24)	2 (66.7%) 1 (33.3%)	10 (100%) 0 (0%)	9 (60%) 6 (40%)	24 (75%) 8 (25%)	7 (43.8%) 9 (56.3%)	
Prognosis ^†^ Recovery (65) Mortality (9)	3 3 (100%) 0 (0%)	10 10 (100%) 0 (0%)	15 9 (60%) 6 (40%)	32 31 (96.9%) 1 (3.1%)	14 12 (85.7%) 2 (14.3%)	0.0076 **

^†^, by Fisher’s exact test; ***, *p* < 0.001; **, *p* < 0.01; *, *p* < 0.05.

**Table 2:** Clinical variables of children and adults with intramural hemorrhage.

Clinical Variables (Case Number)	Children (n = 31)	Adults (n = 49)	*p*-Value
Trauma ^†^ No (38) Yes (10)	19 10 (52.6%) 9 (47.4%)	29 28 (96.6%) 1 (3.4%)	0.0004 ***
Anemia/Pale ^‡^ No (21) Yes (36)	21 4 (19%) 17 (81%)	36 17 (47.2%) 19 (52.8%)	0.0334 *
Prognosis ^†^ Recovery (62) Mortality (9)	30 23 (76.7%) 7 (23.3%)	41 39 (95.1%) 2 (4.9%)	0.0305 *

†, by Fisher’s exact test; ^‡^, by chi-square test; ***, *p* < 0.001; *, *p* < 0.05.

**Table 3:** Clinical variables of recovery and mortality groups.

Clinical Variables (Case Number)	Recovery (n = 68)	Mortality (n = 9)	*p*-Value
Age ^†^ Children (30) Adults (41)	62 23 (37.1%) 39 (62.9%)	9 7 (77.8%) 2 (22.2%)	0.0305 *
Location ^†^ Esophagus (3) Stomach (10) Duodenum (15) Small intestine (32) Colon (14)	65 3 (4.6%) 10 (15.4%) 9 (13.8%) 31 (47.7%) 12 (18.5%)	9 0 (0%) 0 (0%) 6 (66.7%) 1 (11.1%) 2 (22.2%)	0.0076 **
Inhibitor ^†^ No (34) Yes (11)	43 33 (76.7%) 10 (23.3%)	2 1 (50%) 1 (50%)	0.4333
Bleeding into lumen of GI tract ^†^ No (44) Yes (25)	64 41 (64.1%) 23 (35.9%)	5 3 (60%) 2 (40%)	1
Bleeding out of serosa of GI tract ^†^ No (45) Yes (23)	64 44 (68.8%) 20 (31.2%)	4 1 (25%) 3 (75%)	0.1087
Intussusception ^†^ No (64) Yes (10)	65 55 (84.6%) 10 (15.4%)	9 9 (100%) 0 (0%)	0.3466
Management ^†^ Conservative (52) Operation (22)	65 48 (73.8%) 17 (26.2%)	9 4 (44.4%) 5 (55.6%)	0.1149

^†^, by Fisher’s exact test; **, *p* < 0.01; *, *p* < 0.05.

**Table 4:** Cases of intramural hematoma involving the small intestine in patients with hemophilia.

	Age/Gender	Public-Ation Year	Coagulation Disorder/Inhibitor Titer (BU/mL)	Site of Hematoma (Etiology/Complications) ^†^	Symptom and Sign	Lab Data ^‡^	Treatment	Outcome	Reference
**1**	43/male	2022	Severe hemophilia A	Jejunum (impending intussusception)	Generalized abdominal pain, nausea, decreased appetite, tarry stool, rebound tenderness	(1) Hb: 14.9 WBC: 13,170 (2) Hb: 15.0 WBC: 13,690	factor VIII infusion	Symptom resolution	Our case
**2**	29/male	2021	Severe hemophilia A (FVIII: C < 1%)/negative inhibitor	Jejunum (jejuno-jejunal intussusception)	RLQ pain with muscle rigidity, vomiting, and complete ileus, abdominal distension	WBC: 9200 Hb: 14 CRP: 22	factor VIII infusion	Reduction of intussusception, Regression of hematoma	[1]
**3**	23/male	2020	Severe hemophilia A (FVIII: C < 1%)/ negative inhibitor	Small intestine	Generalized abdominal pain, abdominal distension, vomiting, diarrhea, blackish stool, muscle guarding	Hb: 12.2 WBC: 8240	emergency laparotomy (Small bowel resection with end-to-end anastomosis)	Uneventful recovery	[47]
**4**	37/male	2016	Severe hemophilia B (FIX: C < 1%)	Distal small intestine	Abdominal pain, vomiting, abdominal distention	Hb: 13→11	factor VIIa infusions	Symptoms of obstruction resolved	[48]
**5**	20/male	2015	Severe hemophilia A (FVIII: C < 1%)	Jejunum (intestinal subocclusion)	Abdominal pain, nausea, vomiting, fever, abdominal distension	WBC: 9810 Hb: 15.9	factor VIII with von Willebrand factor	Regression of hematoma	[4]
**6**	-/-	2015	Hemophilia A (FVIII < 0.05)	Jejunum	Abdominal distension, nausea, vomiting, ileus	WBC: 11,100 Hb: 16.6 CRP: 68.6	factor VIII infusion	Symptoms of ileus resolved	[2]
**7**	49/male	2014	Severe hemophilia A (FVIII: C < 1%)/ negative inhibitor	Small intestine (bloody fluid in peritoneal cavity, hemorrhagic infarction)	Bilious vomiting, abdominal pain, peritoneal sign, abdominal distention, no diarrhea, no fever	-	surgical intervention (end-to-end anastomosis)	Complete recovery	[49]
**8**	28/male	2013	Severe hemophilia A	Ileum	Abdominal tenderness, occult blood in stool	-	factor VIII infusion	Complete resolution	[50]
**9**	55/male	2013	Severe hemophilia A/negative inhibitor	Jejunum	Epigastric pain, anorexia, nausea, vomiting, signs of peritoneal irritation, intestinal bleeding	Hb: 14.6 WBC: 15,000	factor VIII infusion	Complete resolution	[51]
**10**	25/male	2011	Severe hemophilia A	Ileum	Abdominal pain, vomiting, occult blood in stool	Hb: 15.9 WBC: 10,500	factor VIII infusion	Thickness of the wall had regressed	[52]
**11**	37/male	2010	Hemophilia A	Distal ileum	Generalized abdominal tenderness	Hb: 9.7 WBC: 10,700	conservative therapy	-	[7]
**12**	42/male	2007	Hemophilia A	Small intestine	Abdominal pain	-	conservative therapy	Complete resolution	[53]
**13**	17/male	2006	Severe hemophilia A (FVIII: C < 1%)/high inhibitor (1024 BU/mL)	Jejunum	Total 5 episodes: (1~3) abdominal pain, vomiting (4) abdominal pain, melena, muscular defense (5) abdominal pain, melena	(1) Hb: 14.8 WBC: 9900 (2) Hb:16.8 WBC:12,200 CRP: 8.2	(1~4) APCC (5) rFVIIa → APCC	Symptom resolution	[5]
**14**	34/male	2005	Severe hemophilia A/high inhibitor	Jejunum (acute intestinal obstruction)	Abdominal pain, no passed for 2 d, vomit green bile, abdominal distension	Hb: 16.4 WBC: 18,000	rFVIIa	Symptom resolution	[3]
**15**	74/male	2004	Mild hemophilia A/inhibitor (27 BU/mL)	Jejunum	Hematemesis, severe anemia, signs of bowel obstruction	Hb: 7	aPCC followed by rFVIIa	-	[54]
**16**	54/male	2003	Moderate hemophilia A (FVIII: C 3%)	Small intestine	Abdominal pain, diarrhea, pale conjunctiva, hyperactive bowel sound	Hb: 10.0 WBC: 4700	factor VIII infusion	Symptom resolution	[55]
**17**	38/-	2002	Moderate hemophilia A (FVIII: C 2.7%)	Small intestine	Abdominal pain, vomiting, peritoneal sign, hypoactive bowel sound	Hb: 13.6 WBC: 3500	emergency laparotomy (resected involved bowel and end-to-end anastomosis)	Uneventful recovery	[56]
**18**	14/male	1999	Severe hemophilia A	Jejunum (trauma-related)	Traumatic intramural hematoma	-	factor VIII infusion	Resorption of the hematoma	[57]
**19**	54/male	1998	Mild hemophilia A (FVIII: C 16%)	Jejunum (on the anal side of the Y anastomosis after Roux-en-Y gastrectomy)	Coffee-ground emesis, diffuse abdominal tenderness, decreased bowel sound	Hb: 7.1	second laparotomy (remove intramural blood clot)	Obstructive ileus improved	[58]
**20**	47/male	1995	Severe hemophilia A (FVIII < 1 U/dL)/low titer of inhibitor (0.9 BU/mL)	Terminal ileum	Abdominal pain, subocclusive state mimic appendicitis, blood present in stool	Hb: 14.3	factor VIII infusion	Complete resolution	[59]
**21**	68/male	1991	Severe hemophilia A/no inhibitor	Terminal ileum	RLQ abdominal pain, abdominal distention, diarrhea, peritoneal sign, hypoactive bowel sound	Hct: 39% WBC: 4400	factor VIII infusion	Complete resolution of hematoma	[60]
**22**	49/male	1990	Severe hemophilia A (FVIII: C < 1%)	Small intestine	Generalized abdominal pain, abdominal distention, nausea, no vomiting, decreased appetite	Hb: 9.7	factor VIII infusion	Symptoms subsided	[23]
**23**	31/male	1985	Hemophilia A	Jejunum (500 mL hemoperitoneum)	Angina, abdominal pain, nausea, vomiting	Hb: 16.6 WBC: 17,600	laparotomy (small bowel resection)	-	[61]
**24**	40/male	1985	Hemophilia A	Small intestine	Abdominal pain, vomiting	Hb: 14.1 WBC: 18,600	factor VIII infusion	Symptom resolution	[61]
**25**	8/male	1982	Hemophilia A	Jejunum (trauma-related/ jejuno-jejunal intussusception with mild edematous pancreatitis)	Abdominal pain, nausea, vomiting, epigastric palpable mass, bilateral flank dullness, rebound tenderness, hemodynamic instability	Hb: 8.3 WBC: 19,000 Amylase: 75→725 (56~190)	exploratory laparotomy (jejunal hematoma evacuation, intussusception reduced)	Follow up normal	[42]
**26**	34/male	1978	Hemophilia A/anti-factor VIII antibodies	Small intestine	Epigastric pain, abdominal distension, melena, hematemesis, anemia	Hct: 47→17%	clotting factor, vasopressin infusion	Significant hematemesis and died	[11]
**27**	19/male	1978	Hemophilia A	Small intestine (transient intussusception)	Diffuse abdominal pain, nausea, vomiting, melena, no bowel movement, slightly bloody vomitus, anemia	Hct: 40→23%	clotting factor	Symptom resolution	[11]
**28**	16/male	1978	Hemophilia B	Distal jejunum (intermittent intussusception)	Postprandial vomiting, periumbilical pain, no bowel movement	-	clotting factor	Symptom resolution	[11]
**29**	47/male	1978	Hemophilia	jejunum	RUQ pain with decreased bowel sound, nausea, vomiting, anorexia, bloody stool	Hct: 28→23%	clotting factor	Symptom resolution	[11]
**30**	59/male	1977	Hemophilia A/ anti-factor VIII antibody	Small intestine	Abdominal pain, intra-abdominal irritation, occult blood	Hct: 42.6%	-	-	[62]
**31**	31/male	1976	Hemophilia	Small intestine	Abdominal pain, melena	-	-	-	[63]
**32**	-/-	1973	Hemophilia	ileum	volvulus, intestinal obstruction symptoms	-	surgical resection of ileum	Symptom-free for 2 years	[64]
**33**	16/male	1972	Hemophilia A	Small intestine	Generalized abdominal pain, absolute constipation, abdominal distension, recurrent vomiting, dehydration, anemia, hematemesis, melena	Hb: 9 WBC: 8000	fresh blood and conservative therapy	Condition improved	[65]
**34**	12/male	1967	Hemophilia	Ileum (trauma-related)	Abdominal cramping pain, vomiting, generalized weakness, pale, peritoneal sign	Hb: 6 WBC: 25,900	laparotomy (resection of the intestine with end-to-end anastomosis)	Condition improved	[66]

^†^, if data available; ^‡^, Unit: Hb, hemoglobin (g/dL); WBC, white blood cell count (/μL); CRP, C-reactive protein (mg/dL).

**Table 5:** Cases of intramural hematoma involving the stomach in patients with hemophilia.

	Age/Gender	Public-Ation Year	Coagulation Disorder/Inhibitor Titer (BU/mL)	Site of Hematoma (Etiology/Complications) ^†^	Symptom and Sign	Lab Data ^‡^	Treatment	Outcome	Reference
**1**	24/male	2004	Hemophilia A	Stomach lesser sac	Cramping abdominal pain, nausea	WBC: 11,500 CRP: 4.6	factor VIII infusion	Symptoms subsided	[67]
**2**	10/male	1999	Severe hemophilia A (FVIII: C < 1%)	Stomach lesser sac	Non-colicky hypochondrial pain	-	factor VIII infusion	Symptoms subsided	[68]
**3**	20/male	1988	Hemophilia A	Posterior wall of the stomach and in the lesser sac	Severe abdominal pain, abdominal distension, nausea, vomiting	-	factor VIII infusion	Symptoms subsided	[69]
**4**	5.5 month/male	1988	Moderate hemophilia A (FVIII: C: 1%)	Pylorus (iatrogenic/gastric outlet obstruction)	Signs of gastric outlet obstruction	Hb: 7.3	cryoprecipitate	Widened pyloric lumen	[70]
**5**	23/male	1986	Mild hemophilia A (FVIII: C: 10%)	Stomach	Low-grade fever, cramping abdominal pain, nausea, diarrhea, muscle guarding	Hct: 43→29% WBC: 4200	cryoprecipitate	Resolution of the hematoma	[71]
**6**	9y11m/male	1981	Hemophilia A (factor VIII <0.1 unit/mL)/low titer inhibitor (4~8 BU/mL)	Stomach (partial gastric outlet obstruction)	Cramping intermittent LUQ pain, nausea, early satiety, vomiting with postprandial regurgitation, LUQ firm and tender mass, anemia	Hb: 7.6 Hct: 22% WBC: 11,600	antihemophilic factor (AHF) concentrate	Symptoms subsided	[27]
**7**	23/male	1978	Severe hemophilia A	Stomach	Epigastric pain with LUQ muscle guarding, vomiting, anemia, no bloody stool, no hematuria	Leukocytosis with left shift, Elevated CRP	factor VIII infusion	Symptoms subsided	[72]
**8**	22/male	1974	Hemophilia A	Stomach	Epigastric pain, nausea, vomiting	-	cryoprecipitate anti-hemophilic globulin	Symptoms subsided	[73]
**9**	8/male	1971	Hemophilia A	Stomach	Nausea, vomiting with bile-stained vomitus, left hypochondrial mass	-	antihemophilic globulin	Mass regression	[35]
**10**	8.5/male	1965	Hemophilia	Stomach (significant bleeding into retrogastric space)	Anemia	Hb: 9→6.9	fresh frozen plasma	Resolution of the hematoma	[74]

^†^, if data available; ^‡^, Unit: Hb, hemoglobin (g/dL); WBC, white blood cell count (/μL); CRP, C-reactive protein (mg/dL).

**Table 6:** Cases of intramural hematoma involving the esophagus in patients with hemophilia.

	Age/Gender	Public-Ation Year	Coagulation Disorder/Inhibitor Titer (BU/mL)	Site of Hematoma (Etiology/Complications) ^†^	Symptom and Sign	Lab Data ^‡^	Treatment	Outcome	Reference
**1**	n.s/male	2002	Hemophilia A	Esophagus	Acute odynophagia, substernal chest pain radiating to the anterior chest and shoulder	-	factor VIII infusion	Resolution of symptoms and hematoma	[36]
**2**	31/male	1992	Moderate hemophilia A (basal factor VIII level: 4.5%)/without inhibitor	Esophagus from incisor to carina (hematoma intraluminally ruptured)	Retrosternal pain radiate to back, dysphagia, acute distress and febrile, bloody stool, significant hematemesis	Hb: 7.3 WBC: 14,600	cryoprecipitate with surgical intervention (gastrostomy, esophagostomy)	Resolution of symptoms and hematoma	[75]
**3**	38/male	1977	Hemophilia A	Esophagus	Nausea, hematemesis	Hct: 40→32%	antihemophilic factor	Complete resolution of lesion	[76]

^†^, if data available; ^‡^, Unit: Hb, hemoglobin (g/dL); WBC, white blood cell count (/μL); CRP, C-reactive protein (mg/dL).

**Table 7:** Cases of intramural hematoma involving the duodenum in patients with hemophilia.

	Age/Gender	Public-Ation Year	Coagulation Disorder/Inhibitor Titer (BU/mL)	Site of Hematoma (Etiology/Complications) ^†^	Symptom and Sign	Lab Data ^‡^	Treatment	Outcome	Reference
**1**	52/male	2022	Hemophilia A	Duodenum with acute edematous pancreatitis	Epigastric abdominal pain, vomiting, absence of intestinal transit	Hb: 8.5 WBC: 17,700 CRP: 66.3 (0~3) elevated lipase and amylase	factor VIII infusion	Recovery of pancreatitis and hematoma	[30]
**2**	7/male	2014	Moderate to severe hemophilia B (FIX level: 2%)	Duodenum (intra-peritoneal hemorrhage)	Hemodynamic instability, abdominal pain, abdominal distention, changed mental status, vomiting, anorexia	Severe anemia	exploratory laparotomy (drainage of hematoma)	Uneventful recovery	[31]
**3**	14/male	2014	Mild hemophilia A	Duodenum (significant hemoperitoneum)	Abdominal pain, abdominal distension, hypovolemic shock, peritoneal sign	Hct: 27.4%	emergency laparotomy (evacuation of hematoma)	Uneventful recovery	[77]
**4**	10/male	1996	Hemophilia A	Duodenum (caused gastric outlet obstruction)	Abdominal pain, non-bilious vomiting, constipation, muscle guarding, decrease bowel sound	normal blood counts	factor VIII infusion	Symptoms resolved	[42]
**5**	47/male	1992	Hemophilia	Duodenum	Epigastric pain, right hypochondrium pain, N/V, muscle defense	-	coagulation factor infusion	Symptoms resolved	[78]
**6**	15/male	1990	Mild hemophilia A (FVIII:13.2%)	Duodenum (trauma-related/retroperitoneal bleeding and acute pancreatitis)	Epigastric pain, hypotension with hypovolemic shock, muscle guarding, abdominal distention	Hb: 12.8→8.1 WBC: 27,400 Amylase: 8530	emergency laparotomy (subtotal duodenectomy preserving the pylorus and papillae)	Large amount of bleeding from the drain on 6th post-operation day, improved after factor VIII infusion	[79]
**7**	12/male	1989	Hemophilia A	Duodenum (retroperitoneal bleeding)	Right side abdominal pain, vomiting, pallor, weakness, right iliac tenderness with rebound pain, cholestatic jaundice	Hct: 27% elevated amylase	factor VIII infusion (abdomen was entered initially without definite diagnosis)	Hematoma decreased rapidly and bleeding episode stabilized under conservative treatment	[80]
**8**	child	1988	Hemophilia	Duodenum	-	-	deferred surgery (>24 h)	Died	[20]
**9**	child	1988	Hemophilia	Duodenum	-	-	deferred surgery (>24 h)	Died	[20]
**10**	child	1988	Hemophilia	Duodenum	-	-	conservative therapy	Died	[20]
**11**	child	1988	Hemophilia	Duodenum	-	-	conservative therapy	Died	[20]
**12**	16/male	1988	Hemophilia A with inhibitor	Duodenum	Abdominal pain, vomiting	-	aPCC	Resolution of symptoms	[69]
**13**	18/male	1988	Hemophilia A	Duodenum (acute obstructive pancreatitis)	Acute periumbilical pain, bilious vomiting, diminished bowel sound, palpable mass at RUQ	Hct: 42.9% Amylase: 3000	factor VIII infusion	Resolution of symptoms	[32]
**14**	11/male	1977	Hemophilia	Duodenum (rapidly developing hemomediastinum)	Severe abdominal pain	-	surgical intervention	Died	[24]
**15**	2/male	1975	Severe hemophilia A	Duodenum (trauma-related/obstruction of the opening of ampulla Vater causing dilatation of CBD, significant hemoperitoneum)	Significant hematemesis, dark red vomitus, febrile, drowsy, pale, generalized hypotonia, hypotension, pulsatile and palpable mass in the epigastrium	Hb: 7.6	cryoprecipitate	Died (sudden deterioration)	[22]

^†^, if data available; ^‡^, Unit: Hb, hemoglobin (g/dL); WBC, white blood cell count (/μL); CRP, C-reactive protein (mg/dL).

**Table 8:** Cases of intramural hematoma involving the colon (from the cecum to the rectum) in patients with hemophilia.

	Age/Gender	Public-Ation Year	Coagulation Disorder/Inhibitor Titer (BU/mL)	Site of Hematoma (Comorbidities/Complications) ^†^	Symptom and Sign	Lab Data ^‡^	Treatment	Outcome	Reference
**1**	10/male	2019	Severe hemophilia A	Rectum	Abdominal pain, pelvic tenderness, long-lasting constipation	Hb: 6	-	-	[81]
**2**	15/male	2015	hemophilia A	Cecum (mimic acute appendicitis)	Abdominal pain in the right iliac fossa, vomiting, anorexia, abdominal guarding	Hb: 9.4 WBC: 16,000	exploratory laparotomy (appendectomy and drainage)	Uneventful recovery	[82]
**3**	7/male	2014	Mild hemophilia A /high titer inhibitor(>10 BU/mL)	Sigmoid colon (traumatic Hx)	Rectorrhagia, colicky pain in the suprapubic region	no anemia	rFVII a, aPCC	Resolution of hematoma	[83]
**4**	17/male	2009	Severe hemophilia A without inhibitors	Sigmoid colon (severe cough Hx/ruptured into peritoneal space causing hemoperitoneum and right hematocele)	Swelling of the lower abdomen and right scrotum, abdominal distention with large tender palpable mass in the infraumbilical region, Hb drop	Hb: 11.7→8.7 WBC: 17,800 CRP: 79 (<3)	factor VIII infusion	Regression of hemoperitoneum	[6]
**5**	55/male	2008	Hemophilia A	Sigmoid colon (complete intestinal obstruction)	Right side abdominal pain, abdominal distention, acute compartmental syndrome (renal dysfunction and respiratory distress)	WBC: 22,900 Hb: 9.5 Hct: 27.5%	emergency laparotomy (Hartman procedure)	Uneventful recovery	[29]
**6**	29/male	2007	Severe hemophilia A	1st: Caecum and ascending colon 2nd: Transverse colon (two associated colo-colic intussusceptions)	Right iliac fossa pain, vomiting, large tender and locally guarded mass in the RLQ, rectorrhagia	Hb: 15.1 WBC: 16,400	factor VIII infusion	Reduction in size of the hematoma and complete resolution of the intussusceptions	[28]
**7**	65/male	2006	Hemophilia A/inhibitor: 1 BU/mL	Cecum (ileus → [16 days later] → colo-colic intussusception)	Abdominal distension, right- side abdominal pain → [16 days later] → bloody stool, palpable mass in the RLQ, no rebound tenderness	Hb: 10.1 Hct: 32.9% WBC: 9100	conservative therapy → [16 days later] → laparotomy (surgical reduction fail, followed by right colectomy)	Uneventful recovery	[33]
**8**	26/male	1999	Severe hemophilia A (FVIII: C < 1%)	Rectum	Lower abdominal pain, constipation	-	cryoprecipitate	Symptoms resolved	[84]
**9**	38/male	1990	Moderate hemophilia A (FVIII activity: 2%)	Ascending colon	Abdominal pain with muscle defense, palpable firm mass, no vomiting, no per-anal bleeding	No anemia WBC: 12,100	laparotomy (right hemicolectomy) (because factor VIII infusion failed)	Died of sepsis with multi-organ failure	[23]
**10**	25/male	1987	Hemophilia A with inhibitor	Cecum (ceco-colic intussusception)	Right side severe abdominal pain, abdominal distention, hematochezia, nausea, vomiting, decreased bowel sound	Hct: 20% WBC: 10,000	laparotomy (right hemicolectomy)	Uneventful recovery	[85]
**11**	23/male	1985	Hemophilia B	Cecum (appendix rupture, intraperitoneal abscess)	Abdominal complaints, vomitus, loss of appetite	-	surgery	-	[86]
**12**	12/male	1982	Hemophilia A	Sigmoid colon to rectum	Diffuse abdominal tenderness, pale, peritoneal sign	Hb: 9.6	laparotomy	Died (severe underlying disease)	[21]
**13**	49/male	1979	Hemophilia A	Ascending colon (fistula formed between colon and iliacus muscle)	Appendicitis-like symptoms	-	operation (bypass cecum and ascending colon, with ileum and transverse colon anastomosis)	-	[87]
**14**	13.5/male	1977	Severe hemophilia A (FVIII activity: <1%)	Colon (ileocolic intussusception)	RUQ abdominal pain, vomiting, palpable mass in the RUQ	-	barium enema reduction, cryoprecipitate	Symptoms improved	[41]
**15**	9/male	1977	Hemophilia A	Descending colon	Abdominal pain, melena, severe anemia	Severe anemia	cryoprecipitate	Uneventful recovery	[24]
**16**	36/male	1972	Mild hemophilia A (FVIII concentration 8~19%)	Sigmoid colon (rupture into peritoneal space)	Constipation, abdominal distension, tenderness (maximal in the left iliac fossa), small passage of blood per rectum	Hypovolemia	laparotomy (resected involved sigmoid colon)	Postoperative intraperitoneal hemorrhage episodes resolved under cryoprecipitate	[39]
**17**	12/male	1956	Hemophilia A	Distal transverse colon	Bloody stool, hypotension, pale	Hb: 6.8 Hct: 21% WBC: 6800	fresh frozen plasma	Resolution of hematoma	[88]

^†^, if data available; ^‡^, Unit: Hb, hemoglobin (g/dL); WBC, white blood cell count (/μL); CRP, C-reactive protein (mg/dL).

## Data Availability

Data are available upon reasonable request to the corresponding authors.
